# Potential Probiotic Strains From Milk and Water Kefir Grains in Singapore—Use for Defense Against Enteric Bacterial Pathogens

**DOI:** 10.3389/fmicb.2022.857720

**Published:** 2022-04-01

**Authors:** Li Ling Tan, Chuan Hao Tan, Noele Kai Jing Ng, Yoke Hun Tan, Patricia Lynne Conway, Say Chye Joachim Loo

**Affiliations:** ^1^School of Materials Science and Engineering, Nanyang Technological University, Singapore, Singapore; ^2^Singapore Centre for Environmental Life Sciences Engineering, Nanyang Technological University, Singapore, Singapore; ^3^School of Biological, Earth and Environmental Sciences, The University of New South Wales, Sydney, NSW, Australia; ^4^Lee Kong Chian School of Medicine, Nanyang Technological University, Singapore, Singapore

**Keywords:** kefir, probiotics, screening, pathogen inhibition, competitive, sequencing, safety, Singapore

## Abstract

Kefir grains consist of complex symbiotic mixtures of bacteria and yeasts, and are reported to impart numerous health-boosting properties to milk and water kefir beverages. The objective of this work was to investigate the microbial communities in kefir grains, and explore the possibility of deriving useful probiotic strains from them. A total of 158 microbial strains, representing six fungal and 17 bacterial species, were isolated from milk and water kefir grains collected from a Singapore-based homebrewer. Based on 16S rRNA sequencing, isolated genera included *Lactobacillus*, *Liquorilactobacillus*, *Lacticaseibacillus*, *Lentilactobacillus*, *Leuconostoc*, *Lactococcus*, *Acetobacter*, *Gluconobacter*, *Oenococcus*, *Clostridium*, *Zymomonas*, *Saccharomyces*, *Kluyveromyces*, *Pichia*, *Lachancea*, *Candida*, and *Brettanomyces*. To characterize these isolates, a funnel approach, involving numerous phenotypic and genomic screening assays, was applied to identify kefir-derived microbial strains with the highest probiotic potential. Particular focus was placed on examining the pathogen inhibitory properties of kefir isolates toward enteric pathogens which pose a considerable global health burden. Enteric pathogens tested include species of *Bacillus*, *Salmonella*, *Vibrio*, *Clostridium*, *Klebsiella*, *Escherichia*, and *Staphylococcus*. Well diffusion assays were conducted to determine the propensity of kefir isolates to inhibit growth of enteric pathogens, and a competitive adhesion/exclusion assay was used to determine the ability of kefir isolates to out-compete or exclude attachment of enteric pathogens to Caco-2 cells. Seven bacterial strains of *Lentilactobacillus hilgardii*, *Lacticaseibacillus paracasei*, *Liquorilactobacillus satsumensis*, *Lactobacillus helveticus*, and *Lentilactobacillus kefiri*, were ultimately identified as potential probiotics, and combined to form a “kefir probiotics blend.” Desirable probiotic characteristics, including good survival in acid and bile environments, bile salt hydrolase activity, antioxidant activity, non-cytotoxicity and high adhesion to Caco-2 cells, and a lack of virulence or antimicrobial resistance genes. In addition, vitamin and γ-aminobutyric acid (GABA) synthesis genes, were identified in these kefir isolates. Overall, probiotic candidates derived in this study are well-characterized strains with a good safety profile which can serve as novel agents to combat enteric diseases. These kefir-derived probiotics also add diversity to the existing repertoire of probiotic strains, and may provide consumers with alternative product formats to attain the health benefits of kefir.

## Introduction

The human body is an ecosystem which is home to trillions of microbes. In the recent two decades, international collaborative projects such as the Human Microbiome Project (HMP) (Turnbaugh et al., 2007) and METAgenomics of the Human Intestinal Tract (MetaHIT) (Qin et al., 2010), have brought to light the intricate interactions between the human microbiome and our human health. Dysbiosis of the human gut microbiota, which indicates a state where microbiota richness and diversity is significantly deviated from a healthy control state, has been correlated with numerous gut diseases, and chronic health conditions such as obesity, type II diabetes, immune diseases and neurological disorders (Shreiner et al., 2015). The idea that perturbations of the human microbiome may cause disease sparked considerable interest and the question posed: Could there be healthy microbes supplemented to the human body that reverse the disease state—i.e., probiotics? Because of the promising health benefits of probiotics, interest in these “healthy microbes” has risen rapidly in recent years, with a market estimate projecting a compound annual growth rate (CAGR) of 7% through 2028 (Grand View Research, 2022). Existing commercial probiotic bacterial strains are mostly *Lactobacilli*,^1^ *Streptococcus*, *Bacillus*, *Bifidobacterium*, or *Saccharomyces*, and have been sourced from the native microbiota in human feces, human breast milk as well as from fermented food sources.

One crucial mechanism by which probiotics elicit health benefits is through inhibition of enteric bacterial pathogens. Enteric pathogens are disease-causing microorganisms which infect the gastrointestinal tract, typically leading to symptoms of diarrhea, nausea, abdominal pain, fever or in severe cases, death. Enteric bacterial pathogen-associated diseases are a significant global health burden; in 2010, based on a World Health Organization (WHO) report, enteric bacterial pathogens accounted for 350 million cases of illnesses, 187 thousand deaths and the loss of 5.7 million healthy years of life worldwide (WHO, 2022). Currently, antibiotics are employed as first-line therapy for enteric bacterial infections. However, there is a need to reduce antibiotics usage because of rising instances of antimicrobial resistance (AMR) and the spread of multi-drug resistant organisms. Such concerns have led to a 2019 report by the Centers for Disease Control and Prevention (CDC) declaring that humankind has entered the “post-antibiotic” era (Centers for Disease Control Prevention, 2019), calling for alternative solutions to target enteric bacterial infections. As such, probiotics are now being studied in greater detail for their potential to inhibit and treat enteric bacterial infections.

Kefir is an ancient fermented beverage well-known for its purported health-boosting properties. Originating from the Caucasus mountains, kefir is traditionally produced from kefir grains, which are three-dimensional cauliflower-like granules comprising a symbiotic mixture of bacteria, yeasts, and their extracellular polymeric substances. There are two types of kefir—milk kefir and water kefir, each of them being fermented by placing respective grains in milk or sugary water and incubating at ambient temperature. Bacterial and fungal genera which have been previously identified in kefir include lactobacilli, *Lactococcus*, *Streptococcus*, *Leuconostoc*, *Acetobacter*, *Saccharomyces*, *Kluyveromyces*, and *Candida* (Bourrie et al., 2016; Moretti et al., 2022). The microbial composition of milk and kefir grains are reported to differ slightly (Fiorda et al., 2017). Numerous scientific and clinical studies have taken closer examination of kefir’s nutritional and medicinal efficacies. Health-promoting qualities, such as antipathogenic effects, antioxidant properties, cholesterol assimilation, tumor suppression, and gastrointestinal immunity have been identified (Bourrie et al., 2016; Gut et al., 2021). For instance, several lactobacilli and yeast strains from kefir exhibited antipathogenic activities, inhibiting the growth of *Salmonella typhimurium* and enteropathogenic *Escherichia coli* O157:H7 (Santos et al., 2003; Menezes et al., 2020). *Lactobacillus acidophilus*, *Lactiplantibacillus plantarum*, and *Lentilactobacillus kefiri* strains from milk kefir were found to lower serum total cholesterol, low-density lipoprotein (LDL) and triglyceride levels in Sprague Dawley rats fed with a high cholesterol diet (Zheng et al., 2013). In a study by Vinderola et al. (2005), kefir containing viable microorganisms was found to be more effective in modulating the gut mucosal immune system than pasteurized kefir. These examples suggest that the health-promoting properties of the kefir beverage can be attributed to the properties of strains present within kefir. Herein, the motivation of this study is to identify beneficial probiotic strains from both milk and water kefir grains.

In this paper, microorganism from locally-sourced milk and water kefir grains were isolated and characterized. A series of phenotypic and genotypic screening assays were conducted to identify strains with the highest probiotic potential. These assays include agar well diffusion for detection of antipathogenic effects, acid and bile resistance, bile salt hydrolase (BSH) activity, antioxidant activity, non-cytotoxicity, and adhesion to Caco-2 cells. DNA extracted from the isolates of interest was subjected to whole genome sequencing (WGS). The seven best performing kefir-derived strains were combined in a “kefir probiotic blend” formulation, and a competitive adhesion/exclusion assay of kefir probiotics against enteric pathogens to Caco-2 cells was conducted. Tested human enteric pathogens include *Bacillus cereus*, *Salmonella enterica*, *Vibrio parahaemolyticus*, *E. coli* O157:H7, *Klebsiella pneumoniae*, *Staphylococcus aureus*, *Clostridium difficile*, and *Clostridium perfringens*. Overall, this study explores the potential of using kefir-sourced microbial strains as probiotics to inhibit enteric pathogens, and provides detailed *in vitro* characterization of their safety and other functional properties.

## Materials and Methods

### Materials

Milk and water kefir grains used in this study were active grains donated by a homebrewer in Singapore. *Lacticaseibacillus rhamnosus* GG (LGG) (i-Health Inc., Cromwell, CT, United States) and *L. plantarum* (wild type strain), were used as positive probiotic controls in various assays. *E. coli* K-12, provided by colleagues at SCELSE, was used as a negative control for the well diffusion assay. Enteric pathogens used in this study are detailed in Table 1. All enteric pathogens were purchased from ATCC, Manassas, VA, United States, with exception of *K. pneumoniae* and *S. aureus* which were provided by colleagues at SCELSE. Human intestinal Caco-2 cell lines HTB-37™ were purchased from ATCC, Manassas, VA, United States.

**TABLE 1:** Growth conditions of pathogens.

Pathogen species	Strain	Media	Temperature (°C)	Aerobic/anaerobic (OX/ANA)	Incubation duration (days)
*Bacillus cereus*	ATCC 11778	NB	30	OX	1
*Salmonella enterica*	ATCC-BAA-190	NB	37	OX	1
*Vibrio parahaemolyticus*	ATCC 17802	NB w/3% NaCl	37	OX	1
*Clostridium perfringens*	ATCC 13124	TSB	37	ANA	1
*Clostridium difficile*	ATCC 9689-FZ	TSB	37	ANA	2
*Klebsiella pneumoniae*	KP-1	TSB	37	OX	1
*Escherichia coli* O157:H7	ATCC 43888	TSB	37	OX	1
*Staphylococcus aureus*	USA300	TSB	37	OX	1

*Static conditions were used for incubation of all strains.*

De Man, Rogosa, and Sharpe (MRS), Mannitol-yeast extract peptone (MYP), Yeast Extract-Peptone-Glycerol (YPG), Yeast Extract-Peptone-Dextrose (YPD), M17, and Nutrient broth/agar (NB/NA) media, were used for isolation of strains from the kefir granules. MRS, M17, and NB media were purchased from Thermo Fisher Scientific, Waltham. MA, United States. YPD was purchased from Sigma-Aldrich, St. Louis, MO, United States. MYP medium was prepared with 25 g/l mannitol, 5 g/l yeast extract, 3 g/l peptone (Sievers and Swings, 2005). YPG contained 10 g/l peptone, 5 g/l yeast extract, 0.01 g/l bromophenol blue. 150 μg/ml of cycloheximide were added to MRS, GM and NB media to inhibit yeast growth; 100 mg/l of chloramphenicol was added into YPG and YPD media to inhibit bacteria growth. Antibiotics were filtered and added into respective media after autoclaving. Tryptic soy (TS) medium, used for culturing of enteric pathogens, was purchased from Thermo Fisher Scientific, Waltham. MA, United States. Bacto agar was purchased from BD, Franklin Lakes, NJ, United States. Yeast extract, Yeast protein extract reagent (YPER), 2,2-diphenyl-1-picrylhydrazyl (DPPH), Dulbecco’s Modified Eagle Medium (DMEM), fetal bovine serum (FBS), trypsin-EDTA were purchased from Thermo Fisher Scientific, Waltham. MA, United States. Q5 High Fidelity PCR kit was purchased from New England Biolabs, Ipswich, MA, United States. The CytoTox 96^®^ non-radioactive cytotoxicity kit was purchased from Promega, Madison, WI, United States. DNeasy Ultraclean Microbial Kit was purchased from QIAgen, Hilden, Germany. All other chemicals used in this experiment were purchased from Sigma Aldrich, St. Louis, MO, United States. Sterilization by autoclaving at 121°C for 15 min was done for every media, chemical solutions, or apparatus prior to use, where necessary.

### Methods

#### Isolation of Microbes From Milk and Water Kefir

Milk and water kefir grains were ground with mortar and pestle, then serially diluted in phosphate buffered solution (PBS), before spreading on various media, including MRS, MYP, YPG, YPD, NA, and M17 agar (1.5% agar used). These media were selected in attempt to isolate of a variety of kefir microorganisms; MRS was used to isolate lactobacilli; MYP for acetic acid bacteria (Sievers and Swings, 2005); YPG and YPD for yeast (Martínez-Torres et al., 2017); NA as a general growth medium, and M17, for lactic streptococci (Terzaghi and Sandine, 1975). Plates were incubated at 30°C for 3–7 days at both aerobic (air) and anaerobic (4% H_2_, 5% CO_2_, 91% N_2_) conditions. Anaerobic handling of strains was done within the Bactron 300 anaerobic chamber (Sheldon Manufacturing, Cornelius, OR, United States). Representative colonies were selected for isolation and purified by streaking onto fresh agar plates. Purified colonies were then cultured in corresponding broths and aliquots were stored at –80°C with 20% (v/v) glycerol as cryoprotectant.

#### Species Identification by 16S rRNA and Internal Transcribed Spacer Sequencing

The 16S rRNA and internal transcribed spacer (ITS) segments were sequenced to determine the species identity of kefir-derived microbial strains. Individual isolates were cultured in respective broths and 100 μl of cell cultures were lysed by addition of 20 μl YPER and heating at 98°C for 5 min (Packeiser et al., 2013). The cell lysate was centrifuged at 14,000 × *g* for 10 min, and the supernatant was collected for polymerase chain reaction (PCR). Q5 High Fidelity PCR kit (New England Biolabs, MA, United States) was used with universal primers 27F (5′ AGAGTTTGATCMTGGCTCAG 3′), and 1492R (5′ TACGGYTACCTTGTTACGACTT 3′) to amplify 16S rRNA for bacteria. For yeast, primers used were ITS 1 (5′ TCCGTAGGTGAACCTGCGG 3′) and ITS 4 (5′ TCCTCCGCTTATTGATATGC 3′). The PCR reaction mix consisted of 10 μl 5X Q5 reaction buffer, 1 μl 10 mM dNTPs, 25 μM forward primer, 25 μM reverse primer, 0.5 μl Q5 High Fidelity DNA polymerase, 5 μl DNA template and 28.5 μl nuclease-free water, totaling 50 μl. PCR amplification was carried out with following parameters: 98°C for 3 min, 30 cycles of 98°C for 10 s, 57°C for 15 s, 72°C for 1 min, 72°C for 2 min and holding at 12°C. Quality of PCR products was checked by gel electrophoresis, performed at 100 V for 50 min with ethidium bromide (EtBr) staining for 30 min. Gels were viewed under UV light with Universal Hood II Gel Doc system (Bio-Rad Laboratories, Hercules, CA, United States) to obtain the band image. Samples that produced defined bands were sent to an external vendor (1st base, Singapore) for sequencing. Briefly, PCR products were purified using FavorPrep™ GEL/PCR Purification Kit (Favorgen Biotech Corporation, Pingtung, Taiwan). Purified DNA templates were then subjected to cycle sequencing following standard protocol from BigDye^®^ Terminator v3.1 Cycle Sequencing Kit (Applied Biosystems, Waltham, MA, United States). Products were purified by MagBio HighPrep™ DTR Clean-up System (Magbio Genomics, Gaithersburg, MD, United States) before loading into ABI 3730xl DNA Analyzer (Applied Biosystems, Waltham, MA, United States) for DNA Sanger Sequencing. Obtained nucleotide sequences were analyzed using the ApE plasmid editor software (Davis, 2022) and species assignment of kefir isolates was done using the National Centre for Biotechnology Information (NCBI) BLAST platform, based on the BLAST result which yielded highest total score.

#### Agar Well Diffusion Assay

Antimicrobial activities of isolates from kefir strains against pathogens were assessed using agar well diffusion (Ayala et al., 2019). Pathogen cultures were inoculated in respective media and growth conditions as described in Table 1, before adjustment to OD600 of 0.1 (except KP1, which was adjusted to 0.01), and spreading 100 μl onto the agar plates. Kefir isolates were cultured in MRS broth at 30°C, anaerobic conditions for 3 days. Thirty-six strains representing 23 species were selected for this assay. LGG, cultured in MRS at 37 °C, aerobic conditions for 24 h, was used as a positive probiotic control. Wells 6 mm in diameter were formed in the agar and filled with 50 μl of cultures (contains microbes) or culture supernatants (without microbes), with duplicates performed for each kefir isolate strain. Cell-free supernatants were obtained by centrifuging the incubated cultures at 10,000 × *g* for 5 min. Plates were incubated in growth conditions according to the growth requirements of each pathogen. Finally, plates were observed for the presence of inhibition zones around individual wells. Inhibition zones are clear sites that have no visible pathogen growth, indicating that the kefir isolates have successfully inhibited growth of the enteric pathogen.

#### Acid and Bile Resistance

The protocol of Chandel et al. (2019) was used to examine the acid and bile resistance of the kefir isolates to determine their survival capacity during exposure to acidic pH and bile typical of the human GIT. In this assay, MRS broths were adjusted to pH 2, pH 3, or with 0.3% (w/v) ox-bile added. Aliquots (100 μl) of cultures of the kefir isolate were added to 4.9 ml of each of the adjusted MRS broths in duplicates and incubated at 37°C for 2 h under 200 rpm shaking conditions in an Ecotron shaking incubator (Infors AG, Bottmingen, Switzerland). Ten-fold serial dilutions and drop plating (100 μl aliquots) was done to enumerate the colony-forming units (CFU/ml) before and after acid/bile exposure.

#### Bile Salt Hydrolase Activity Assay

Bile salt hydrolase activity is a desirable probiotic trait which has been postulated to increase survival and persistence of microbes in the intestinal tract, and has been reported to also induce cholesterol-lowering efficacies by controlling serum cholesterol levels (Begley et al., 2005). BSH activities of kefir isolates were evaluated as previously reported by Michael et al. (2016). Aliquots (5 μl) of the cultures of the kefir isolates were inoculated onto MRS agar containing 0.5% (w/v) taurodeoxycholate hydrate (TDC) in duplicates. Plates were incubated at 37°C under anaerobic conditions for 24 h. BSH activity was indicated by the development of a white precipitate on TDC-supplemented plates after 48 h. *L. plantarum* was used as a positive control in this assay (Michael et al., 2016).

#### Antioxidant Activity Assay

Antioxidant activities of kefir isolates were determined using the DPPH free radical assay (Kedare and Singh, 2011). Aliquots (0.5 ml) of cultures of kefir isolates were added to 3 ml of 0.05 mM DPPH in ethanol in duplicate. Controls were prepared by mixing MRS broth (0.5 ml) with 3 ml of absolute ethanol. The reaction mixture was then incubated in the dark at room temperature for 30 min. A color change from deep violet to light yellow could be observed if antioxidant activity was present. After incubation, the absorbance at 517 nm was measured with a spectrophotometer. The antioxidant activity percentage (AA%) was determined according to:


(1) $$\mathrm{AA\%}=[1-(A_{\mathrm{sample}}/A_{\mathrm{control}})],$$

where A_sample_ is the average of measured absorbance at 517 nm of the sample with DPPH added minus the absorbance of MRS broth without DPPH added, and A_control_ is the measured absorbance at 517 nm of MRS broth with DPPH added minus the absorbance of MRS broth without DPPH added.

#### Cytotoxicity of Kefir Isolates Using Caco-2

The *in vitro* cytotoxicity of kefir isolates was determined using Caco-2 cells. The Caco-2 cells were maintained in DMEM containing 20% (v/v) FBS, 100 units/ml of penicillin and 100 μg/ml of streptomycin, at 37°C in a humidified 5% CO_2_ atmosphere. Caco-2 cells were seeded at a concentration of 2.8 × 10^4^ cells/cm^2^ in 24-well tissue culture plates and grown for 7 days until confluence was reached. For the last medium change, DMEM without antibiotics was used. Duplicate wells with confluent Caco-2 cells were inoculated with 0.5 ml of 1 × 10^7^ CFU/ml (as determined by optical density from a standard culture of OD and CFU) kefir isolates, which were prepared by washing respective cultures of kefir isolates in PBS and resuspending in DMEM. Inoculated Caco-2 plates were then incubated for 24 h in 37°C, 5% CO_2_ conditions. Following incubation, Caco-2 cells were washed twice with PBS, and the number of Caco-2 cells that remained viable in each well was determined with the CytoTox 96^®^ non-radioactive cytotoxicity kit, using a protocol from Van den Bossche et al. (2020) which reduces bacterial interference with lactate dehydrogenase (LDH) quantification. Caco-2 cells were completely lysed with 200 μl of 1× Lysis solution (from kit) and incubated at 37°C for 45 min. Efficient lysis of cells was further achieved through vigorous pipetting. Lysed Caco-2 cells were then centrifuged at 5,000 × *g* for 10 min, and 50 μl of the supernatant was added to 50 μl CytoTox 96^®^ Reagent. Following a 30-min incubation at room temperature in dark conditions, 50 μl of Stop Solution (from kit) was added to each sample, and absorbance at 490 nm was measured in a microplate reader (Infinite M200, Tecan Group Ltd, Männedorf, Switzerland). Un-inoculated Caco-2 wells were used as maximum lysis controls, and 1× Lysis solution was used to determine the background absorbance. Background absorbance was subtracted from all measured absorbance values with Caco-2 cells. Percent cytotoxicity was calculated as follows:


$$\begin{array}{c} \%\mathrm{cytotoxicity}=\\ \;100-100*\frac{\mathrm{LDH}\mathrm{from}\mathrm{remaining}\mathrm{viable}\mathrm{cell}\mathrm{fraction}}{\mathrm{Maximum}\mathrm{LDH}\mathrm{release}\mathrm{from}\mathrm{no}\mathrm{bacteria}\mathrm{control}} \end{array}$$

#### Adhesion of Kefir Isolates to Caco-2

The ability of kefir isolates to adhere to an intestinal surface was determined by an *in vitro* adhesion assay using the human epithelial cell line Caco-2 (Ayala et al., 2019). Caco-2 cells were seeded at 2.8 × 10^4^ cells/cm^2^ in 12-well tissue culture plates and the culture medium was changed daily for 21 days to allow growth into the late post-confluence stage. For the last medium change, DMEM without antibiotics was used. Duplicate confluent Caco-2 cell monolayers were inoculated with 1 ml of 1 × 10^8^ CFU/ml kefir isolates, which were prepared by washing cultures of the kefir isolates in PBS and re-suspending in DMEM. Inoculated Caco-2 plates were then incubated for 2 h in 37°C, 5% CO_2_ conditions to allow for microbial attachment. Following incubation, non-attached or loosely adherent microbes were removed by washing Caco-2 monolayers three times with sterile PBS. To release adherent microbes, 200 μl of 0.25% (w/v) trypsin with 0.53 mM EDTA were added to each well, and incubated for 10 min in 37°C, 5% CO_2_ conditions. PBS (800 μl) was then added per well to dilute the trypsin-EDTA, and ten-fold serial dilutions and drop-plating was done to enumerate CFU of attached kefir microbes. Percentage adhesion was calculated by taking the ratio of attached microbes to the CFU of microbes added.

#### Whole Genome Sequencing and Genotypic Characterization

Selected kefir isolates were genotypically characterized by WGS. Genomic DNA was extracted from kefir isolates using a DNeasy Ultraclean Microbial Kit, according to manufacturer’s instructions. The quality and concentration of extracted DNA concentration was checked *via* gel electrophoresis and a Qubit 2.0 Fluorometer (Thermo Fisher Scientific, Waltham. MA, United States), respectively, prior to sequencing. Library preparation was performed using a TruSeq DNA HT Library Preparation Kit (Illumina, San Diego, CA, United States), and sequencing was done on the Illumina MiSeq platform with 300 base pairs (bp) paired end reads. Raw reads were cleaned using Trimmomatic version 0.39 (Bolger et al., 2014), and quality of reads was checked with FastQC (Babraham Bioinformatics, 2022). *De novo* genome assembly was done using SPAdes version 3.14.1 (Bankevich et al., 2012), and the quality of resultant assembled contigs was checked with the DDBJ Fast Annotation and Submission Tool (DFAST) (Tanizawa et al., 2016). DFAST was also used to confirm the species and taxonomy of kefir isolates *via* average nucleotide identity (ANI) analysis (Jain et al., 2018). Assembled contigs of kefir isolates Kef-w/m1-21 were submitted to GenBank under the BioProject ID PRJNA721546, with accession numbers JAGPZD000000000 to JAGPZX000000000. Functional gene annotation of assembled contigs was performed using the NCBI prokaryotic genome annotation pipeline (PGAP). Bacteriocins, virulence factors and AMR genes were identified using the BAGEL4 (Van Heel et al., 2018), Virulence Finder v2.0.3 (Joensen et al., 2014), and ResFinder 4.1 pipelines (Bortolaia et al., 2020) respectively. Plasmids were identified using PlasmidFinder 2.1 (Carattoli et al., 2014). Biogenic amines (BAs) and toxins production genes (including genes related to production of histidine decarboxylase, tyrosine decarboxylase, ornithine decarboxylase, agmatine dehydrolase, l-lysine decarboxylase, agmatine deiminase, hemolysin, cytotoxin, fengygin, surfactin, lychenisin, and lipopolysaccharides) were manually searched for among annotated genes of each kefir isolate. Vitamins and γ-aminobutyric acid (GABA) biosynthesis genes were also searched for manually. Taxonomic analysis was performed using the Type (Strain) Genome Server (TYGS), a free bioinformatics platform available under https://tygs.dsmz.de (Meier-Kolthoff and Göker, 2019). Briefly, the kefir isolate genomes were compared against all type strain genomes available in the TYGS database *via* the MASH algorithm to determine closely related type strains. These strains were compared pairwise to derive their intergenomic distances, then used to infer a balanced minimum evolution tree with branch support *via* FASTME 2.1.6.1 including SPR post-processing.

#### Preparation of Kefir “Probiotic Blend”

Based on the above phenotypic and genotypic characterization assays, seven best performing kefir bacterial strains were selected for combination in a multi-strain “kefir probiotic blend” (Table 2). The kefir probiotics blend was prepared by adjusting the cultures of the selected kefir isolates to indicated OD600 values and concentrating each of the isolates into the same volume by centrifugation and resuspending in PBS. Total CFU/ml of kefir probiotics blend was approximately 3 × 10^9^ CFU/ml.

**TABLE 2:** Composition of kefir “probiotics blend.”

Kefir probiotic species	Strain	OD600 used in kefir probiotic blend	CFU in 1 ml of kefir probiotic blend	CFU ratio
*Lentilactobacillus hilgardii*	Kef-w9	5.2	6.03E+07	0.02
*Lacticaseibacillus paracasei*	Kef-w14	6	8.57E+08	0.28
*Lacticaseibacillus paracasei*	Kef-w19	6	8.25E+08	0.27
*Liquorilactobacillus satsumensis*	Kef-w13	5.6	4.13E+08	0.13
*Liquorilactobacillus satsumensis*	Kef-w11	5.6	5.56E+08	0.18
*Lactobacillus helveticus*	Kef-m4	6	1.75E+08	0.06
*Lentilactobacillus kefiri*	Kef-m15	2.5	2.06E+08	0.07

#### Competitive Adhesion/Exclusion of Enteric Pathogens by the Kefir Probiotic Blend

An important probiotic mechanism of action is through adhesion to host cells, which competitively blocks the adherence of, or excludes, pathogens from host cell binding sites (Monteagudo-Mera et al., 2019). The ability of kefir isolates to compete for adhesion sites with or exclude pathogens from Caco-2 epithelial cells, was tested using a modification of an established method (Candela et al., 2008; Inturri et al., 2016). Briefly, Caco-2 cells were seeded at a concentration of 2.8 × 10^4^ cells/cm^2^ in 12-well tissue culture plates and grown for 21 days to allow growth into the late post-confluence stage. For the last medium change, DMEM without antibiotics was used. Cultures of the eight human enteric pathogens included in Table 1 and the kefir probiotics blend as indicated in Table 2 were used. For this assay, pathogens and kefir isolates were prepared by washing cultures in PBS and re-suspending in DMEM, to yield 1 × 10^8^ CFU/ml respectively. Duplicate confluent Caco-2 cells were inoculated with either: (1) 1 ml of a mixture of kefir isolates and individual pathogens, and incubated for 2 h, for the competitive adhesion assay, or (2) 1 ml of kefir isolates, incubated for the first hour, prior to the removal of kefir isolates and the addition of 1 ml of individual pathogens and incubated for the next hour, for the competitive exclusion assay. Incubation was done under 37°C, 5% CO_2_ conditions to allow for probiotics/pathogens attachment. Following incubation, non-attached or loosely adherent bacteria were removed by washing Caco-2 monolayers three times with sterile PBS. To release adherent bacteria, 200 μl of trypsin-EDTA were added to each well, and incubated for 10 min in 37°C, 5% CO_2_ conditions. PBS (800 μl per well) was then added to dilute the trypsin-EDTA, and drop-plating of aliquots from ten-fold serial dilutions onto selective agars (e.g., NA or TS media were used to enumerate enteric pathogens, while MRS was used to enumerate kefir isolates) to enumerate attached probiotics/pathogens as CFU. The rationale of the competitive adhesion method is to determine if the kefir isolates could reduce pathogen adhesion to Caco-2 when added in combination, while competitive exclusion determines if pre-exposure to kefir microbes can reduce attachment of pathogens. The CFU data of attached probiotics/pathogens in the competitive adhesion and exclusion methods were hence compared to the adhesion data of individual pathogens to Caco-2 cells. Normalized adhesion was calculated as the percentage of pathogens adhered (competitive adhesion or competitive exclusion method) divided by the percentage of pathogens adhered when added alone.

#### Statistical Analysis

All experiments were performed at least in duplicate. Results for various assays were expressed as the mean of duplicates, with the relevant standard deviation data provided in Supplementary Table 1. Statistical evaluation was conducted for results of the competitive exclusion/adhesion assay, and one way analysis of variance and *post-hoc* Dunnett test was used. Letters on bars were based on *p < 0.05, ^**^p < 0.01, ^***^p < 0.001, ^****^p < 0.0001, ns–no significant difference.

## Results

### List of Isolated Strains

In total, 158 strains representing 6 fungal and 17 bacterial species, were isolated from milk and water kefir (Table 3). Isolated genera include *Lactobacillus*, *Liquorilactobacillus*, *Lacticaseibacillus*, *Lentilactobacillus*, *Leuconostoc*, *Lactococcus*, *Acetobacter*, *Gluconobacter*, *Oenococcus*, *Clostridium*, *Zymomonas*, *Saccharomyces*, *Kluyveromyces*, *Pichia*, *Lachancea*, *Candida*, and *Brettanomyces*. Most species were isolated from milk or water kefir exclusively, with exception of *Pichia fermentans*, *Saccharomyces cerevisiae*, and *Clostridium beijerinckii*, which were isolated from both milk and water kefir.

**TABLE 3:** List of microbial strains isolated from milk and water kefir.

No.	Microorganism Species	Kingdom	Number of strains isolated	Strain names	Source	Isolation media	Previously isolated from kefir	Previously identified in kefir by metagenomic techniques
1	*Liquorilactobacillus satsumensis*	Bacteria	20	Kef-w1, Kef-w2, Kef-w11, Kef-w13, Kef-w18, Kef-w22-36	Water Kefir	MRS, MYP, NA, YPD, M17	Miguel et al., 2011; Zanirati et al., 2015; Gamba et al., 2019	Kumar et al., 2021
2	*Lactobacillus kefiranofaciens*	Bacteria	4	Kef-m3, Kef-m37-39	Milk Kefir	MRS, MYP	Santos et al., 2003	Nalbantoglu et al., 2014; Korsak et al., 2015; Chen et al., 2021
3	*Lactobacillus helveticus*	Bacteria	1	Kef-m4	Milk Kefir	MYP	Simova et al., 2002; Miguel et al., 2011	Nalbantoglu et al., 2014; Chen et al., 2021
4	*Acetobacter persici*	Bacteria	4	Kef-w5, Kef-w40-42	Water Kefir	MYP	Brandt et al., 2017	-
5	*Leuconostoc mesenteroides*	Bacteria	5	Kef-m6, Kef-m16, Kef-m43-45	Milk Kefir	MRS, M17	Pidoux, 1989; Gulitz et al., 2011; Zanirati et al., 2015	Nalbantoglu et al., 2014; Korsak et al., 2015, Kazou et al., 2021
6	*Lacticaseibacillus paracasei*	Bacteria	12	Kef-w7, Kef-w14, Kef-w17, Kef-w19-20, Kef-w46-52	Water Kefir	MRS, NA, YPD, M17	Santos et al., 2003; Miguel et al., 2011; Gamba et al., 2019; Talib et al., 2019	Korsak et al., 2015; Verce et al., 2019; Chen et al., 2021
7	*Lentilactobacillus hilgardii*	Bacteria	3	Kef-w8-10	Water Kefir	MRS	Pidoux, 1989; Gamba et al., 2019	Cao et al., 2019; Verce et al., 2019; Kumar et al., 2021
8	*Zymomonas mobilis*	Bacteria	10	Kef-w12, Kef-w53-61	Water Kefir	MRS, YPD, YPG	-	Cao et al., 2019
9	*Lentilactobacillus kefiri*	Bacteria	2	Kef-m15, Kef-m62	Milk Kefir	MRS, YPD	Gao et al., 2012; Zheng et al., 2013; Zanirati et al., 2015; Hurtado-Romero et al., 2021	Nalbantoglu et al., 2014; Korsak et al., 2015; Kazou et al., 2021
10	*Liquorilactobacillus nagelii*	Bacteria	1	Kef-w21	Water Kefir	YPD	Gamba et al., 2019	Verce et al., 2019
11	*Lactobacillus delbrueckii*	Bacteria	1	Kef-m63	Milk Kefir	MYP	Simova et al., 2002	Kazou et al., 2021
12	*Lactococcus lactis*	Bacteria	31	Kef-m64-93	Milk Kefir	MRS, MYP, NA, YPD, M17	Simova et al., 2002; Gao et al., 2012; Zanirati et al., 2015; Hurtado-Romero et al., 2021	Nalbantoglu et al., 2014; Korsak et al., 2015
13	*Acetobacter fabarum*	Bacteria	7	Kef-m94-100	Milk Kefir	MRS, MYP, YPD	Gulitz et al., 2011; Gao et al., 2012	-
14	*Kluyveromyces marxianus*	Fungi	8	Kef-m101-108	Milk Kefir	MYP, YPG	Wyder et al., 1997; Simova et al., 2002; Gao et al., 2012; Hsu and Chou, 2021	Chen et al., 2021; Kazou et al., 2021
15	*Acetobacter orientalis*	Bacteria	3	Kef-m109-111	Milk Kefir	MYP, YPD	Gulitz et al., 2011	Korsak et al., 2015
16	*Pichia fermentans*	Fungi	3	Kef-m112-113, Kef-w114	Milk and Water Kefir	YPG	Miguel et al., 2011	-
17	*Saccharomyces cerevisiae*	Fungi	19	Kef-m115, Kef-w116-125, Kef-m126-129, Kef-w130-133	Milk and Water Kefir	YPD, YPG	Simova et al., 2002; Miguel et al., 2011; Gao et al., 2012; Gamba et al., 2019; Hsu and Chou, 2021	Verce et al., 2019; Kazou et al., 2021
18	*Clostridium beijerinckii*	Bacteria	3	Kef-w134, Kef-m135-136	Milk and Water Kefir	MYP, YPD	-	Chen et al., 2021
19	*Lachancea fermentati*	Fungi	11	Kef-w137-147	Water Kefir	YPD, YPG	Gulitz et al., 2011	Marsh et al., 2013
20	*Candida ethanolica*	Fungi	4	Kef-w148-151	Water Kefir	YPG	-	Sarikkha et al., 2015
21	*Gluconobacter oxydans*	Bacteria	1	Kef-w152	Water Kefir	YPD	Gamba et al., 2019	Kumar et al., 2021
22	*Oenococcus oeni*	Bacteria	3	Kef-w153-155	Water Kefir	MYP	Zanirati et al., 2015	Nalbantoglu et al., 2014; Kumar et al., 2021
23	*Brettanomyces anomalus*	Fungi	3	Kef-w156-158	Water Kefir	YPG	Wyder et al., 1997	-

### Pathogen Inhibition Using Well Diffusion Assay

Amongst the 158 kefir-derived isolates, 23 strains representing the 23 distinct species isolated, together with 13 more strains selected to test for strain-specific activity, a total of 36 strains, were selected for a detailed screening. Of these 36 tested strains, 24 kefir-isolates showed inhibitory activities towards at least one of the tested pathogens (Table 4). Photos of agar wells with inhibition zones are included in Supplementary Figure 1. These kefir-isolates were from the *Lactobacillus*, *Liquorilactobacillus*, *Lentilactobacillus*, *Lacticaseibacillus*, *Leuconostoc*, *Lactococcus*, and *Gluconobacter* genera. None of the tested kefir-fungal strains showed antimicrobial properties. The growth of the common food borne pathogens *V. parahaemolyticus*, *B. cereus*, and *S. enterica* were most inhibited by multiple kefir isolates. Inhibition of *C. difficile*, a bacterium which causes diarrhea and colon inflammation in humans (Lessa et al., 2015), was observed for the isolates *Lacticaseibacillus paracasei*, *Liquorilactobacillus satsumensis*, and LGG. Enteropathogenic *E. coli* O157:H7, a particular virulent serotype of *E. coli* known to cause diarrhea, abdominal cramps and other complications (WHO, 2022), was found to be inhibited by several *L. paracasei* strains. *K. pneumoniae* and *S. aureus* were also inhibited by *L. satsumensis*, *L. paracasei*, and *Liquorilactobacillus hilgardii* kefir isolates. The kefir isolates inhibited both Gram-positive (*B. cereus*, *C. difficile*, *S. aureus*) and Gram-negative (*V. parahaemolyticus*, *S. enterica*, *E. coli*, *K. pneumoniae*) pathogens. Inhibitory mechanisms of kefir-isolates against these pathogens are most likely attributed to production of antimicrobial metabolites, such as organic acids, hydrogen peroxide and bacteriocins (Šušković et al., 2010). Notably, the pathogen inhibition capacity of several kefir isolates, particularly *L. paracasei* and *L. satsumensis*, outperformed the reference probiotic LGG. Comparing between different strains of the same species, some slight differences in inhibitory activities can be observed. Overall, *L. satsumensis, Lactobacillus helveticus*, *Leuconostoc mesentorides*, *L. paracasei*, *L. hilgardii*, *L. kefiri*, *Liquorilactobacillus nagelii*, *L. paracasei*, and *Gluconobacter oxydans* showed most promising antimicrobial efficacies. These promising strains were selected for further phenotypic screening.

**TABLE 4:**
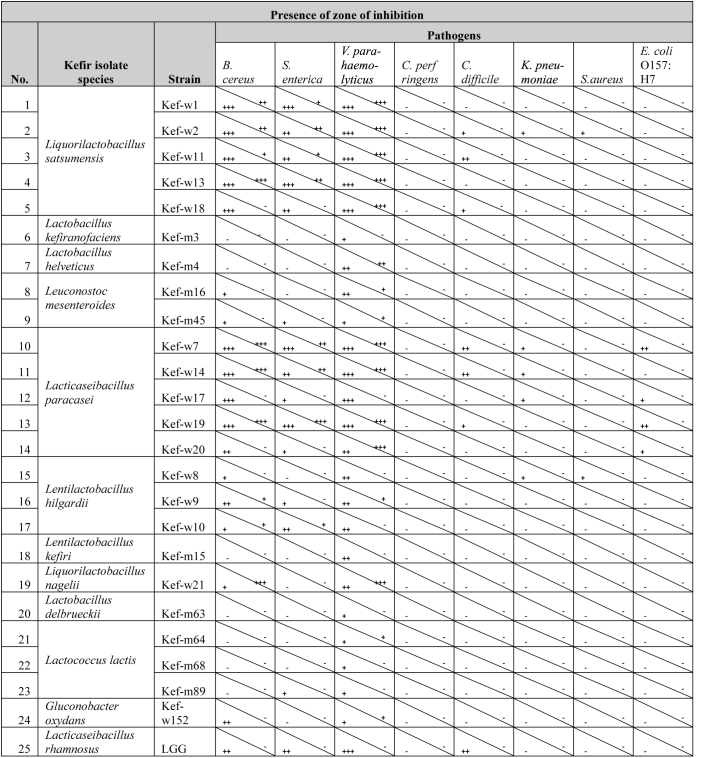
Inhibitory activities of kefir isolates and *Lacticaseibacillus rhamnosus* GG (LGG) against human enteric pathogens.^a^

*^a^The bottom-left triangle indicates inhibition by kefir isolate cultures, while the top-right triangle indicates inhibition by the cell-free supernatant component. Inhibition zones of >4, 2–4, and <2 mm were reviewed as strong (+++), intermediate (++), and weak inhibition (+), respectively. Cells indicated with (-) are without any zone of inhibition observed. Only kefir isolates with inhibitory activities towards at least one tested pathogen are listed. Zones of inhibition were averaged from duplicate wells.*

### Phenotypic Characteristics of Kefir Isolates

From the well diffusion assay results, 24 kefir isolates were selected for further screening and results are presented in Table 5. For acid/bile tolerance, all selected kefir-isolates showed susceptibility to pH 2, similar to the control probiotic LGG. Twelve strains, namely *L. satsumensis* Kef-w1, Kef-w2, Kef-w13, *L. helveticus* Kef-m4, *L. paracasei* Kef-w7, Kef-w14, Kef-w17, Kef-w19, *L. hilgardii* Kef-w8-10, *L. kefiri* Kef-m15 showed the ability to survive 2-h exposure to pH 3 and 0.3% (w/v) ox-bile with <1 logCFU reduction, suggesting their intrinsic resistances to acid and bile exposure typical of the conditions in the human GIT. For BSH activity, seven kefir isolates of species *L. hilgardii*, *L. kefiri*, *Lactobacillus delbrueckii*, and *Lactobacillus lactis* demonstrated BSH activity. Antioxidant activities, ranging from 23 to 78%, were observed of the kefir isolates. Highest antioxidant activity was observed of *L. paracasei* Kef-w19, notably higher than that of the reference strain LGG (Mu et al., 2018). Adhesion of tested kefir isolates to Caco-2 cells were notably higher than that of LGG, measured at 0.9%. The adhesion of kefir isolates ranged from 1.2 to 18.1%, with *L. satsumensis* Kef-w11, *L. hilgardii* Kef-w8-10, *L. kefiri* Kef-m15, and *L. nagelii* Kef-w21 having adhesion percentages of more than 10%, indicative of good adhesion properties (Candela et al., 2005). All tested kefir isolates were found to be non-cytotoxic to Caco-2 cells, showing percentage cytotoxicities of <1.21%.

**TABLE 5:** Phenotypic properties of selected kefir isolates, including results for acid and bile resistance, bile salt hydrolase (BSH) activity, antioxidant activity, adhesion, and cytotoxicity to Caco-2 cells.*^a^*

No.	Kefir isolate species	Strain	Acid and bile resistance, Log_10_CFU surviving				BSH act-ivity	Anti-oxidant activity (AA %)	% Adhered to Caco-2 (%)	Cyto-toxicity to Caco-2 (%)
			Initial	After pH2 exposure	After pH3 exposure	After bile exposure				
1		Kef-w1	7.55	0.00	7.54	6.70	-	56%	7.6%	0.53%
2		Kef-w2	7.51	0.00	7.53	6.83	-	51%	1.2%	–0.37%
3	*Liquorilactobacillus*	Kef-w11	7.70	0.00	7.57	5.28	-	32%	10.0%	–0.05%
4	*satsumensis*	Kef-w13	7.44	0.00	7.31	7.11	-	58%	3.3%	–0.20%
5		Kef-w18	7.55	2.74	7.55	5.54	-	62%	8.9%	1.21%
6	*Lactobacillus kefiranofaciens*	Kef-m3	6.58	3.22	4.11	6.42	-	Nil	Nil	Nil
7	*Lactobacillus helveticus*	Kef-m4	7.37	3.35	7.24	6.58	-	60%	1.2%	0.81%
8	*Leuconostoc mesenteroides*	Kef-m16	6.10	2.74	3.59	5.82	-	Nil	Nil	Nil
9	Kef-m45	6.90	0.00	2.74	6.44	-	Nil	Nil	Nil
10	*Lacticaseibacillus paracasei*	Kef-w7	8.02	0.00	7.74	8.21	-	56%	4.0%	–0.66%
11	Kef-w14	6.32	3.22	6.36	5.85	-	68%	4.8%	0.23%
12	Kef-w17	8.03	0.00	8.09	7.86	-	63%	3.0%	0.07%
13	Kef-w19	7.49	0.00	7.61	7.43	-	78%	5.4%	0.03%
14	Kef-w20	6.90	0.00	5.76	7.09	-	Nil	Nil	Nil
15	*Lentilactobacillus hilgardii*	Kef-w8	7.49	0.00	7.14	7.48	-	23%	17.6%	–2.14%
16	Kef-w9	7.69	0.00	7.12	7.89	+	37%	17.2%	–3.24%
17	Kef-w10	7.64	0.00	7.02	7.48	+	37%	13.3%	–1.62%
18	*Lentilactobacillus kefiri*	Kef-m15	7.28	0.00	6.92	8.24	+	69%	10.2%	0.81%
19	*Liquorilactobacillus nagelii*	Kef-w21	7.58	0.00	7.29	5.35	-	72%	18.1%	1.11%
20	*Lactobacillus delbrueckii*	Kef-m63	6.70	0.00	2.74	6.43	+	Nil	Nil	Nil
21	*Lactococcus lactis*	Kef-m64	6.49	0.00	0.00	6.43	+	Nil	Nil	Nil
22	Kef-m68	7.14	0.00	0.00	6.86	+	Nil	Nil	Nil
23	Kef-m89	7.59	0.00	0.00	7.29	+	Nil	Nil	Nil
24	*Gluconobacter oxydans*	Kef-w152	5.86	0.00	4.91	5.60	-	Nil	Nil	Nil
25	*Lacticaseibacillus rhamnosus*	LGG	7.51	0.00	7.48	7.47	-	73%	0.9%	0.84%
26	*Lactiplantibacillus plantarum*	Wild Type	Nil				+	Nil	Nil	Nil
										

*^a^All results were calculated as an average of duplicate experiments. Nil indicates that the kefir isolate was not studied for the particular assay.*

### Genotypic Characteristics of Kefir Isolates

**FIGURE 1:**
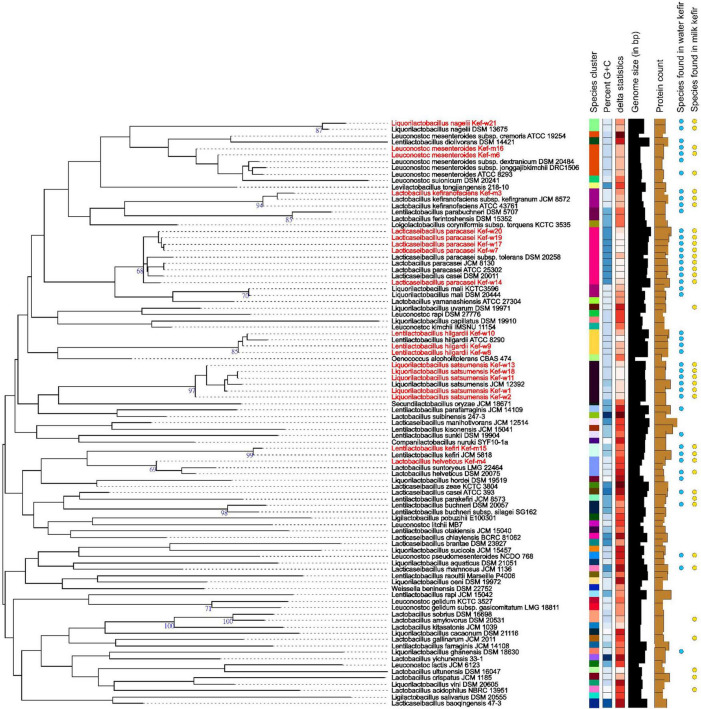
Phylogenetic tree of selected kefir isolates (highlighted in red) and other related strains. The branch lengths are scaled in terms of Genome BLAST Distance Phylogeny approach (GBDP) distance formula *d*_5_.

WGS was carried out for 15 selected kefir isolates, and the collated data of bacteriocins, virulence, antimicrobial resistance (AMR), biogenic amines (BAs), toxins, vitamins, γ-aminobutyric acid (GABA) genes and plasmids for each kefir isolate are presented in Table 6. Antibiotic resistance and virulence genes were absent for all kefir isolates. Plasmids, previously identified in *L. plantarum* (UniProt, 2022) and *Lentilactobacillus buchneri* (Liu et al., 2011), were found present in *L. satsumensis* Kef-w2, Kef-w13, *L. helveticus* Kef-m4 and *L. kefiri* Kef-m15. Genes encoding for production of bacteriocins, which are antimicrobial peptides produced by bacteria and serve as natural alternatives to antibiotics, were identified in *L. satsumensis* Kef-w13, *L. helveticus* Kef-m4, *L. paracasei* Kef-w7, Kef-w14, Kef-w17 and Kef-w19. The toxin protein, hemolysin III, a common hemolysin found in lactobacilli bacteria, was found in all kefir isolates. There are numerous reports about hemolysin III and its safety in lactobacilli, hence this toxin protein is not considered of significant concern (Senan et al., 2015; Surachat et al., 2017). *L. nagelii* Kef-w21 was found to harbor lipopolysaccharide (LPS) endotoxin proteins, which are potentially harmful as LPS can trigger intestinal inflammation and increased permeability. LPS are generally known to be present in Gram-negative bacteria, and the only Gram-positive bacteria so far known to contain an LPS is *Listeria monocytogenes* (Casey et al., 2014). To err on the safe side, *L. nagelii* Kef-w21 was excluded from the kefir probiotic blend. The production of BAs was investigated, as lactobacilli, particularly those involved in fermented foods, often produce them. Notably, synthesis genes associated with histamine and tyramine, two most concerning BAs which have higher incidences of intolerance upon ingestion (Durak-Dados et al., 2020), were found to be absent in the kefir isolates. Agmatine decarboxylase and ornithine decarboxylase genes, which are involved in the biosynthesis of putrescine, were identified in the kefir isolates. Since agmatine decarboxylase and ornithine decarboxylase genes have been previously been identified in many known probiotics and starter cultures (Costantini et al., 2013; Barbieri et al., 2019), this was not considered to be a major safety concern. All kefir isolates were found to contain genes for biosynthesis of riboflavin (vitamin B2), *L. helveticus*, *L. paracasei*, *L. hilgardii* were found to additionally contain cobalamin (vitamin B12) synthesis genes and *L. hilgardii* was found to also contain pyridoxal (vitamin B6) synthesis genes. Genes for biosynthesis of GABA, a neurotransmitter which reduces anxiety and stress (Komatsuzaki et al., 2005), was identified in *L. hilgardii* strains, consistent with other reports (Lee et al., 2013). Phylogenetic analysis revealed several highly similar strains amongst the kefir isolates (Figure 1). *L. paracasei* Kef-w7, Kef-17, Kef-w19, Kef-w20 were highly similar to each other while *L. satsumensis* Kef-w11 and Kef-w18 were similar to each other. Accordingly, only one isolate of very similar strains was selected for the kefir probiotic blend formulation. Ultimately, seven kefir isolates (Table 2) were selected to constitute the kefir probiotic blend based on the phenotypic and genotypic data analyses.

**TABLE 6:** Genetic features of selected kefir isolates.

No.	Kefir isolate species	Strain	AMR genes	Virulence genes	Plasmids	Bacteriocin genes	Toxin synthesis genes	BA synthesis genes	Vitamin synthesis genes	GABA syn-thesis genes
1		Kef-w1	Absent	Absent	Absent	0	Hemolysin III protein	Ornithine decarb-oxylase	Riboflavin	Absent
2		Kef-w2	Absent	Absent	CP-005948	0	Hemolysin III protein	Ornithine decarb-oxylase	Riboflavin	Absent
3	*Liquorilactobacillus satsumensis*	Kef-w11	Absent	Absent	Absent	0	Hemolysin III protein	Ornithine decarb-oxylase	Riboflavin	Absent
4		Kef-w13	Absent	Absent	CP-005948	1	Hemolysin III protein	Ornithine decarb-oxylase	Riboflavin	Absent
5		Kef-w18	Absent	Absent	Absent	0	Hemolysin III protein	Ornithine decarb-oxylase	Riboflavin	Absent
6	*Lactobacillus helveticus*	Kef-m4	Absent	Absent	CP-002655, CP-002654	5	Hemolysin III protein	Ornithine decarb-oxylase	Riboflavin, Cobalamin	Absent
7	*Lacticaseibacillus paracasei*	Kef-w7	Absent	Absent	Absent	2	Hemolysin III protein	Ornithine decarb-oxylase	Riboflavin, Cobalamin	Absent
8	Kef-w14	Absent	Absent	Absent	4	Hemolysin III protein	Ornithine decarb-oxylase	Riboflavin, Cobalamin	Absent
9	Kef-w17	Absent	Absent	Absent	2	Hemolysin III protein	Ornithine decarb-oxylase	Riboflavin, Cobalamin	Absent
10	Kef-w19	Absent	Absent	Absent	2	Hemolysin III protein	Ornithine decarb-oxylase	Riboflavin, Cobalamin	Absent
11	*Lentilactobacillus hilgardii*	Kef-w8	Absent	Absent	Absent	0	Hemolysin III protein	Agmatine deiminase	Riboflavin, Cobalamin, Pyridoxal	Present
12	Kef-w9	Absent	Absent	Absent	0	Hemolysin III protein	Agmatine deiminase	Riboflavin, Cobalamin, Pyridoxal	Present
13	Kef-w10	Absent	Absent	Absent	0	Hemolysin III protein	Agmatine deiminase	Riboflavin, Cobalamin, Pyridoxal	Present
14	*Lentilactobacillus kefiri*	Kef-m15	Absent	Absent	CP-002654	0	Hemolysin III protein	Agmatine deiminase	Riboflavin	Absent
15	*Liquorilactobacillus nagelii*	Kef-w21	Absent	Absent	Absent	0	Hemolysin III protein, LPS protein	Ornithine decarb-oxylase	Riboflavin	Absent
16	*Lacticaseibacillus rhamnosus*	LGG	Absent	Absent	Absent	1	Absent	Ornithine decarb-oxylase	Riboflavin, Cobalamin	Absent

### Competitive Adhesion/Exclusion

**FIGURE 2:**
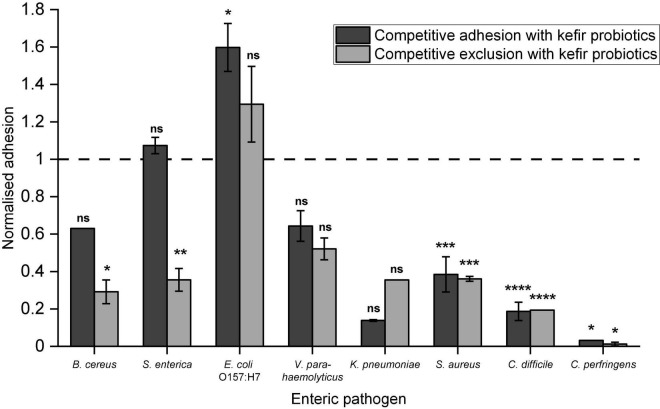
Ability of kefir probiotics to: (a) compete with enteric pathogens for adhesion sites (referred to as competitive exclusion) or (b) exclude adhesion of enteric pathogens when introduced before exposure to the pathogen (referred to as competitive exclusion). Normalized adhesion of 1 indicates that the number of pathogens adhered in competitive adhesion/exclusion with kefir probiotics, was the same as when the pathogens were added alone. Data are presented as an average of duplicates with standard deviation bars. Letters on bars were determined by Dunnett test based on a pairwise-comparison with pathogen-only treatment (refer to Supplementary Table 2 for statistical data). Letters on bars were based on *p < 0.05, **p < 0.01, ***p < 0.001, ****p < 0.0001, ns – no significant difference.

The kefir probiotics blend demonstrated reduction in pathogen adhesion to Caco-2 cells for seven of the eight tested human enteric pathogens, except *E. coli* O157:H7 (Figure 2). Statistically significant reduction in pathogen adhesion was observed for *B. cereus*, *S. enterica*, *S. aureus*, *C. difficile*, and *C. perfringens*. Highest reduction in pathogen adhesion was observed for *C. perfringens*, in which <0.05%, indicating around 3.5 log CFU reduction, of *C. perfringens* cells adhered when kefir probiotics were administered in combination. Notably, a significant reduction in pathogen adhesion (>50%) was also observed for *S. aureus* and *C. difficile*. In comparison with LGG (Supplementary Figure 2), the kefir probiotics blend showed greater inhibition of adhesion of *V. parahaemolyticus* and *C. perfringens*, and similar inhibition of adhesion of the other enteric pathogens. Competitive exclusion appeared to reduce pathogen adhesion more effectively than competitive adhesion, especially for pathogens *B. cereus* and *S. enterica*. This suggests that pre-consumption of kefir probiotics may prophylactically protect epithelial cells against pathogen adhesion better than consumption at the time of exposure to the pathogen.

## Discussion

Bacteria and fungi were isolated from locally-sourced milk and water kefir grains. Isolates were identified to the species level. This process yielded 158 isolates representing 23 microbial species, indicating the wide diversity of culturable microbes from both milk and water kefir. Most of these species have been previously reported to be isolated from kefir, while several were only identified in kefir by culture independent techniques. Notably, *Zymomonas mobilis*, *C. beijerinckii*, and *Candida ethanolica* have not been previously isolated from kefir, but have been purified from beer, soil, vinegar, and apple surfaces, respectively (Richards and Corbey, 1974; Bevardi et al., 2013; Little et al., 2015; Xing et al., 2018; Lynch et al., 2021). While most of these species have been identified in other kefir from different geographical origins, it is worth noting that the microbial species derived in this study were not completely identical to any prior publications. This may be due to differing culture techniques used for the isolation of the microbes from the granules. For instance, Miguel et al. (2011) used only MRS and malt yeast glucose peptone media to isolate bacteria and yeasts, respectively, under aerobic conditions, while in this study, MRS, MYC, NA and M17 media were used for bacteria isolation, YPG and YPD for yeasts isolation, under both aerobic and anaerobic conditions. Inherent differences in microbiota composition in kefir grains of different origins also explains the unique microbiota profile observed in this study. Microbial compositions within kefir grains are known to change between geographical regions, owing to differences in temperature, humidity, altitude conditions, water quality, type of milk/sugar available and hygiene conditions (Zhong et al., 2016). Singapore has a tropical climate, with year-round temperatures ranging from 24 to 32°C. As such, “room temperature” here is considerably higher and more consistent than other climates, which may give rise to microbiota differences in the complex community of the kefir granules. Findings in this study can prove valuable for comparison with studies of the diversity of the kefir microbiota from different geographic origins. Overall, the initial library of 158 strains provided a substantial sample size for this study to proceed, and various assays were conducted to identify the most promising probiotic candidates.

This study adopted a funnel approach to identify kefir-derived microbial strains with highest probiotic potential. Through a series of screening assays, including antimicrobial well diffusion, acid and bile resistance, BSH activity, antioxidant activity, cytotoxicity, adhesion to Caco-2 and whole genome sequencing (WGS), the seven best performing kefir isolates (Table 2) were identified from the initial repertoire of 158. These seven kefir isolates showed good survival [i.e., <2 log(CFU) reduction] in pH 3 acid and bile solutions, non-cytotoxicity and good adhesion to Caco-2, suggesting their suitability for use as oral probiotics. Whole genome analysis revealed an absence of AMR genes and virulence genes, corroborating the safety of these potential probiotic strains. Other qualities identified in the kefir isolates, including BSH activity, high antioxidant activity, presence of vitamins and GABA synthesis genes, add potential functionality of these strains in for general well-being, cholesterol management and mental health aspects. Since these kefir isolates are lactobacilli, and are also isolated from a fermented food source with a safe history of use, these can likely be considered Generally Regarded as Safe (GRAS) by the United States Food and Drug Administration (FDA) (U.S. Food & Drug Administration, 2022), or have Qualified Presumption of Safety (QPS) status granted by the European Food Safety Authority (EFSA) (Koutsoumanis et al., 2019), facilitating their use as potentially novel probiotic agents.

These kefir isolates also demonstrated potent antimicrobial effects against the enteric pathogens *B. cereus*, *S. enterica*, *V. parahaemolyticus*, *E. coli* O157:H7, and *C. difficile*, as demonstrated using the well diffusion assay. Generally, larger zones of inhibition were observed for the wells containing cultures of the kefir isolates, as compared to the cell-free supernatant of the same isolates (Table 4). This suggests that the activity of live cells contributed to the anti-pathogenic property of the kefir isolates, possibly due to additional production of antimicrobial compounds during the period of incubation. From WGS results, bacteriocin genes were identified in various kefir isolate strains, suggesting that bacteriocins production may have been the reason for strong pathogen inhibitory activity demonstrated by these kefir isolates. The seven kefir isolates applied in combination showed the ability to outcompete pathogens *B. cereus*, *S. enterica*, *V. parahaemolyticus*, *K. pneumoniae*, *S. aureus*, *C. difficile*, and *C. perfringens* for epithelial cell adhesion sites and can potentially reduce pathogenesis of these pathogens. It is known that adhesion to intestinal lining plays an important role in pathogenesis of the tested pathogen species (McKee and O’Brien, 1995; Maroncle et al., 2002; Haque et al., 2004; Ritchie et al., 2012; Misawa et al., 2015; Redondo et al., 2015; Tsilia et al., 2015; Anonye et al., 2019). Some of the reduction in pathogen adhesion may also be attributed to the growth inhibitory effects noted for the kefir isolates. As described, enteric bacterial diseases pose a significant global health burden and are an urgent problem to be tackled (WHO, 2022). In particular, non-typhoidal *S. enterica* and enteropathogenic *E. coli*, both which were found to be inhibited by kefir isolates, accounted for >50% of enteric pathogen associated deaths (WHO, 2022). Other tested enteric pathogens, including *B. cereus* [causes 63,400 foodborne disease cases per year in United States alone (Carroll et al., 2019)], *V. parahaemolyticus* [leading cause of seafood-associated infections in United States and Japan (Todd, 2014)], *C. perfringens* [second most frequent cause of bacterial foodborne outbreaks in United States (Todd, 2014)] and *C. difficile* [caused an estimated half a million infections and 29 000 deaths in 2012 in United States (Lessa et al., 2015)], also pose major health concerns and urgently require treatment and prophylactic solutions. The demonstrated impact of the kefir isolates against these pathogens suggests that kefir-derived probiotics are alternative, non-antibiotic, naturally derived candidates for prophylactic and therapeutic applications targeting these enteric pathogens. Further studies will be required to validate the effectiveness of kefir probiotics against enteric bacterial diseases, including examining the effects of kefir probiotics on the intestinal epithelial barrier, investigating the *in vivo* persistence of kefir probiotics in human gut and *in vivo* inhibition of the pathogens in model systems.

The findings from this study can be used to develop alternative means to reap the health benefits of kefir. Kefir is a well-known superfood with numerous purported beneficial qualities, including antipathogenic effects, antioxidant properties, cholesterol assimilation, tumor suppression, and gastrointestinal immunity (Bourrie et al., 2016). Currently, consumption of kefir is primarily *via* drinking the kefir beverage itself, while kefir-derived supplements and functional food products have yet to commercialize extensively. In this study, probiotic candidates isolated from kefir were found to exhibit several of the beneficial properties associated with the kefir beverage itself, such as enteric pathogen inhibition effects, antioxidant properties and some potential for cholesterol management. The probiotic isolates from kefir were also shown to be safe and could be consistently produced through batch fermentations. This suggests a potential avenue for kefir probiotics to be supplemented directly in other product formats to consumers, yet allowing them to receive the desired health benefits. Some of these formats include dietary supplements, such as capsules, tablets, or sachets, or within functional foods and beverages to enhance their nutritional profile. Kefir probiotics may also be encapsulated in polymeric matrices, to imbue additional functional qualities such as protection from the rigors of the GIT and shelf-life stability. These alternative product formats may provide consumers with a greater variety and convenience to attain the health benefits of kefir, thereby facilitating consumers to incorporate diverse microbiome-modulating products into their diet regime.

## Conclusion

Microbes were isolated from Singapore-sourced kefir grains, and through a series of screening assays, seven bacterial isolates with the highest probiotic potential were identified. These kefir isolates demonstrate desirable probiotic characteristics, including good survival in acid and bile environments, BSH activity, antioxidant activity, non-cytotoxicity, and high adhesion to Caco-2 cells, lack of virulence or AMR genes. Notably, kefir isolates also demonstrate antimicrobial activity against enteric pathogens in the well diffusion assay, and showed the capacity to out-compete or exclude the attachment of enteric pathogens to Caco-2 cells. Overall, these kefir isolates represent novel probiotic candidates which can mitigate the burden of enteric pathogen associated diseases and benefit human health. Further studies *via in vitro* and *in vivo* models and clinical studies are required to ascertain the safety and efficacy of these kefir-derived probiotics.

## Data Availability Statement

The datasets presented in this study can be found in online repositories. The link to the datasets can be found at doi: 10.21979/N9/9TG8JH.

## Author Contributions

LT planned and conducted all experiments and analyses and also drafted the manuscript. NN conducted the isolation and species identification of microbes from milk and water kefir. YT assisted in the phenotypic screening assays. CT and SL conceived the project direction. CT, PC, and SL provided advice and guidance on this project and also revised the manuscript. All authors contributed to the article and approved the submitted version.

## Conflict of Interest

The authors declare that the research was conducted in the absence of any commercial or financial relationships that could be construed as a potential conflict of interest.

## Publisher’s Note

All claims expressed in this article are solely those of the authors and do not necessarily represent those of their affiliated organizations, or those of the publisher, the editors and the reviewers. Any product that may be evaluated in this article, or claim that may be made by its manufacturer, is not guaranteed or endorsed by the publisher.

## Footnotes

1 Note that lactobacilli is a generic term to designate all organisms that were classified as *Lactobacillaceae* until 2020 (Zheng et al., 2020).
