# Supplementary material for: Potential Probiotic Strains From Milk and Water Kefir Grains in Singapore—Use for Defense Against Enteric Bacterial Pathogens
Source: Front Microbiol. 2022 Apr 1;13:857720. doi: 10.3389/fmicb.2022.857720 (PMC9011154; doi:10.3389/fmicb.2022.857720)
Supplement: Supplementary file 1 [file Table_1.DOCX]

Supplementary Material


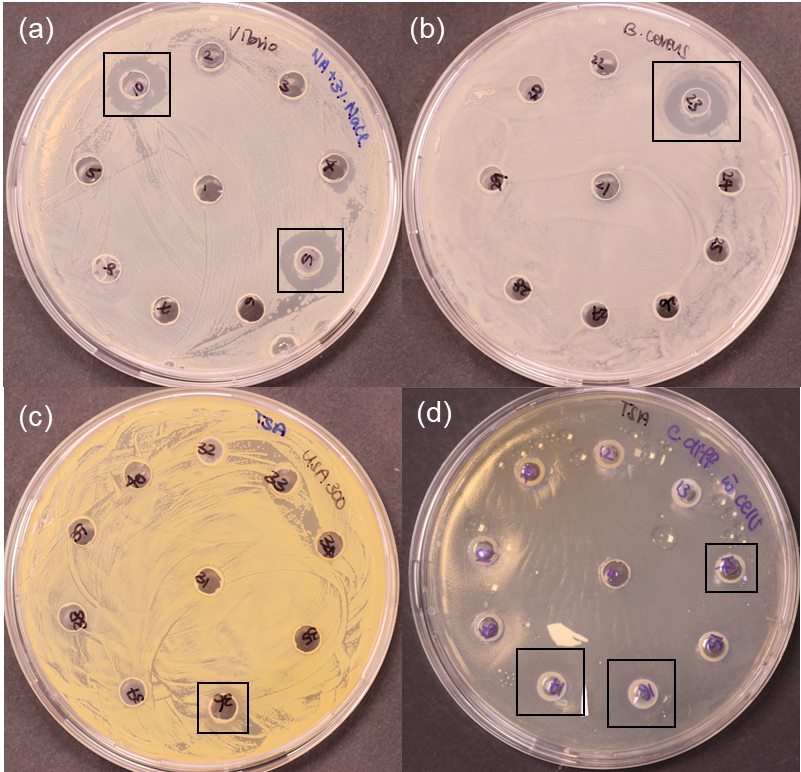


Supplementary Figure S1: Image of various agars with (a) *V. parahaemolyticus*, (b) *B. cereus*, (c) *S. aureus* and (d) *C. difficile* in the agar well diffusion assay. Black boxes indicate that cultures/cell-free supernatants within indicated wells that demonstrated inhibitory activity towards the enteric pathogens.

Supplementary Table S1: Antioxidant activity, adhesion and cytotoxicity to Caco-2 cells of selected kefir isolates. All results were presented as an average of duplicate experiments with standard deviation as indicated.

| **No.** | **Kefir isolate species** | **Strain** | **Antioxidant activity (AA%)** | | **% adhered to Caco-2 (%)** | | **Cyto-toxicity to Caco-2 (%)** | |
| --- | --- | --- | --- | --- | --- | --- | --- | --- |
|  |  |  | **Average** | **Stdev** | **Average** | **Stdev** | **Average** | **Stdev** |
| 1 | *Liquorilactobacillus satsumensis* | Kef-w1 | 56% | 22% | 7.59% | 0.04% | 0.53% | 0.92% |
| 2 |  | Kef-w2 | 51% | 9% | 1.24% | 0.58% | -0.37% | 1.05% |
| 3 |  | Kef-w11 | 32% | 27% | 10.00% | 3.26% | -0.05% | 0.30% |
| 4 |  | Kef-w13 | 58% | 25% | 3.27% | 0.96% | -0.20% | 1.12% |
| 5 |  | Kef-w18 | 62% | 19% | 8.86% | 3.54% | 1.21% | 0.37% |
| 6 | *Lactobacillus helveticus* | Kef-m4 | 60% | 13% | 1.18% | 0.31% | 0.81% | 0.13% |
| 7 | *Lacticaseibacillus paracasei* | Kef-w7 | 56% | 16% | 3.98% | 1.11% | -0.66% | 0.29% |
| 8 |  | Kef-w14 | 68% | 8% | 4.85% | 1.85% | 0.23% | 0.30% |
| 9 |  | Kef-w17 | 63% | 20% | 3.03% | 0.42% | 0.07% | 0.00% |
| 10 |  | Kef-w19 | 78% | 6% | 5.38% | 0.22% | 0.03% | 0.13% |
| 11 | *Lentilactobacillus hilgardii* | Kef-w8 | 23% | 33% | 17.65% | 5.82% | -2.14% | 3.25% |
| 12 |  | Kef-w9 | 37% | 30% | 17.22% | 10.74% | -3.24% | 2.35% |
| 13 |  | Kef-w10 | 37% | 17% | 13.33% | 6.48% | -1.62% | 0.51% |
| 14 | *Lentilactobacillus kefiri* | Kef-m15 | 69% | 6% | 10.22% | 1.54% | 0.81% | 1.59% |
| 15 | *Liquorilactobacillus nagelii* | Kef-w21 | 72% | 8% | 18.08% | 2.72% | 1.11% | 0.10% |
| 16 | *Lacticaseibacillus rhamnosus* | LGG | 73% | 3% | 0.91% | 0.20% | 0.84% | 0.88% |

Supplementary Figure S2: Ability of LGG to: (a) compete with enteric pathogens for adhesion sites (referred to as competitive exclusion) or (b) exclude adhesion of enteric pathogens when introduced before exposure to the pathogen (referred to as competitive exclusion). Normalized adhesion of 1 indicates that the number of pathogens adhered in competitive adhesion/exclusion with kefir probiotics, was the same as when the pathogens were added alone. Data are presented as an average of duplicates with standard deviation bars. Letters on bars were determined by Dunnett test based on a pairwise-comparison with the pathogen-only treatment. * p < 0.05, ** p < 0.01, *** p < 0.001, **** p < 0.0001, ns – no significant difference.

Supplementary Table S2: Statistical analysis of competitive adhesion/exclusion assay.

| **No.** | **Pathogen strain** | **Probiotic treatment** | **Average % adhesion** | **Stdev % adhesion** | **Dunnett's comparison test** | **Mean Diff.** | **95% CI of diff.** | **Adjusted P-value** | **Significance level** |
| --- | --- | --- | --- | --- | --- | --- | --- | --- | --- |
| 1 | *Bacillus cereus* ATCC 11778 | - | 0.41% | 0.08% | - | - | - | - | - |
| 2 |  | LGG, competitive adhesion | 0.14% | 0.09% | Pathogen only VS. LGG, c.a. | 0.270% | 0.07827 to 0.4617 | 0.0131 | * |
| 3 |  | LGG, competitive exclusion | 0.10% | 0.01% | Pathogen only VS. LGG, c.e. | 0.310% | 0.1183 to 0.5017 | 0.0073 | ** |
| 4 |  | Kefir probiotics blend, competitive adhesion | 0.26% | 0.00% | Pathogen only VS. Kefir, c.a. | 0.150% | -0.04205 to 0.3414 | 0.1142 | ns |
| 5 |  | Kefir probiotics blend, competitive exclusion | 0.12% | 0.03% | Pathogen only VS. Kefir, c.e. | 0.287% | 0.0951 to 0.4786 | 0.0102 | * |
| 6 | *Salmonella enterica* ATCC-BAA-190 | - | 1.02% | 0.24% | - | - | - | - | - |
| 7 |  | LGG, competitive adhesion | 0.94% | 0.01% | Pathogen only VS. LGG, c.a. | 0.083% | -0.3045 to 0.4712 | 0.8634 | ns |
| 8 |  | LGG, competitive exclusion | 0.59% | 0.03% | Pathogen only VS. LGG, c.e. | 0.426% | 0.03806 to 0.8138 | 0.0354 | * |
| 9 |  | Kefir probiotics blend, competitive adhesion | 1.09% | 0.04% | Pathogen only VS. Kefir, c.a. | 0.075% | 0.4627 to 0.313 | 0.8984 | ns |
| 10 |  | Kefir probiotics blend, competitive exclusion | 0.36% | 0.06% | Pathogen only VS. Kefir, c.e. | 0.656% | 0.2685 to 1.044 | 0.0060 | ** |
| 11 | *Escherichia coli* O157:H7 ATCC 43888 | - | 0.13% | 0.01% | - | - | - | - | - |
| 12 |  | LGG, competitive adhesion | 0.19% | 0.02% | Pathogen only VS. LGG, c.a. | 0.061% | -0.1241 to 0.0027 | 0.0584 | ns |
| 13 |  | LGG, competitive exclusion | 0.08% | 0.02% | Pathogen only VS. LGG, c.e. | 0.057% | -0.006271 to 0.1206 | 0.0719 | ns |
| 14 |  | Kefir probiotics blend, competitive adhesion | 0.21% | 0.02% | Pathogen only VS. Kefir, c.a. | 0.079% | -0.1424 to -0.01555 | 0.0217 | * |
| 15 |  | Kefir probiotics blend, competitive exclusion | 0.17% | 0.03% | Pathogen only VS. Kefir, c.e. | 0.039% | -0.1023 to 0.02452 | 0.2222 | ns |
| 16 | *Vibrio para-haemolyticus* ATCC 17802 | - | 5.70% | 0.42% | - | - | - | - | - |
| 17 |  | LGG, competitive adhesion | 8.51% | 2.41% | Pathogen only VS. LGG, c.a. | 2.808% | -7.041 to 1.424 | 0.1817 | ns |
| 18 |  | LGG, competitive exclusion | 10.96% | 1.05% | Pathogen only VS. LGG, c.e. | 5.255% | -9.487 to -1.023 | 0.0219 | * |
| 19 |  | Kefir probiotics blend, competitive adhesion | 3.67% | 0.47% | Pathogen only VS. Kefir, c.a. | 2.035% | -2.197 to 6.267 | 0.3748 | ns |
| 20 |  | Kefir probiotics blend, competitive exclusion | 2.97% | 0.33% | Pathogen only VS. Kefir, c.e. | 2.731% | -1.501 to 6.963 | 0.1954 | ns |
| 21 | *Klebsiella pneumoniae* KP-1 | - | 0.01% | 0.01% | - | - | - | - | - |
| 22 |  | LGG, competitive adhesion | 0.00% | 0.00% | Pathogen only VS. LGG, c.a. | 0.011% | -0.004644 to 0.02688 | 0.1538 | ns |
| 23 |  | LGG, competitive exclusion | 0.01% | 0.00% | Pathogen only VS. LGG, c.e. | 0.008% | -0.007997 to 0.02353 | 0.3581 | ns |
| 24 |  | Kefir probiotics blend, competitive adhesion | 0.00% | 0.00% | Pathogen only VS. Kefir, c.a. | 0.012% | -0.003599 to 0.02793 | 0.1183 | ns |
| 25 |  | Kefir probiotics blend, competitive exclusion | 0.01% | 0.00% | Pathogen only VS. Kefir, c.e. | 0.009% | -0.006652 to 0.02487 | 0.2559 | ns |
| 26 | *Staphylococ-cus aureus* USA300 | - | 10.00% | 0.51% | - | - | - | - | - |
| 27 |  | LGG, competitive adhesion | 8.57% | 0.00% | Pathogen only VS. LGG, c.a. | 1.429% | -0.2475 to 3.105 | 0.0865 | ns |
| 28 |  | LGG, competitive exclusion | 2.48% | 0.03% | Pathogen only VS. LGG, c.e. | 7.518% | 5.842 to 9.194 | 0.0001 | **** |
| 29 |  | Kefir probiotics blend, competitive adhesion | 3.85% | 0.94% | Pathogen only VS. Kefir, c.a. | 6.154% | 4.478 to 7.83 | 0.0002 | *** |
| 30 |  | Kefir probiotics blend, competitive exclusion | 3.61% | 0.13% | Pathogen only VS. Kefir, c.e. | 6.393% | 4.717 to 8.069 | 0.0002 | *** |
| 31 | *Clostridium difficile* ATCC 9689-FZ | - | 0.48% | 0.03% | - | - | - | - | - |
| 32 |  | LGG, competitive adhesion | 0.12% | 0.03% | Pathogen only VS. LGG, c.a. | 0.361% | 0.2903 to 0.4322 | 0.0001 | **** |
| 33 |  | LGG, competitive exclusion | 0.03% | 0.00% | Pathogen only VS. LGG, c.e. | 0.451% | 0.3805 to 0.5224 | 0.0001 | **** |
| 34 |  | Kefir probiotics blend, competitive adhesion | 0.09% | 0.02% | Pathogen only VS. Kefir, c.a. | 0.391% | 0.3205 to 0.4624 | 0.0001 | **** |
| 35 |  | Kefir probiotics blend, competitive exclusion | 0.09% | 0.00% | Pathogen only VS. Kefir, c.e. | 0.388% | 0.3171 to 0.459 | 0.0001 | **** |
| 36 | *Clostridium perfringens* ATCC 13124 | - | 65.71% | 32.32% | - | - | - | - | - |
| 37 |  | LGG, competitive adhesion | 30.00% | 14.14% | Pathogen only VS. LGG, c.a. | 35.714% | -19.29 to 90.68 | 0.1920 | ns |
| 38 |  | LGG, competitive exclusion | 10.14% | 2.22% | Pathogen only VS. LGG, c.e. | 55.571% | 0.6019 to 110.5 | 0.0481 | * |
| 39 |  | Kefir probiotics blend, competitive adhesion | 2.09% | 0.00% | Pathogen only VS. Kefir, c.a. | 63.619% | 8.65 to 118.6 | 0.0289 | * |
| 40 |  | Kefir probiotics blend, competitive exclusion | 0.82% | 0.64% | Pathogen only VS. Kefir, c.e. | 64.896% | 9.926 to 119.9 | 0.0268 | * |
